# Future changes in coastal upwelling ecosystems with global warming: The case of the California Current System

**DOI:** 10.1038/s41598-018-21247-7

**Published:** 2018-02-12

**Authors:** Peng Xiu, Fei Chai, Enrique N. Curchitser, Frederic S. Castruccio

**Affiliations:** 1 State Key Laboratory of Tropical Oceanography, South China Sea Institute of Oceanology, Chinese Academy of Sciences, Guangzhou, 510301 China; 2 State Key Laboratory of Satellite Ocean Environment Dynamics, Second Institute of Oceanography, State Oceanic Administration, Hangzhou, 310012 China; 3 School of Marine Sciences, University of Maine, Orono, ME 04469 USA; 4 Department of Environmental Sciences, Rutgers University, New Brunwick, NJ 08901 USA; 5 Climate and Global Dynamics Laboratory, National Center for Atmospheric Research, Boulder, CO 80305 USA

## Abstract

Coastal upwelling ecosystems are among the most productive ecosystems in the world, meaning that their response to climate change is of critical importance. Our understanding of climate change impacts on marine ecosystems is largely limited to the open ocean, mainly because coastal upwelling is poorly reproduced by current earth system models. Here, a high-resolution model is used to examine the response of nutrients and plankton dynamics to future climate change in the California Current System (CCS). The results show increased upwelling intensity associated with stronger alongshore winds in the coastal region, and enhanced upper-ocean stratification in both the CCS and open ocean. Warming of the open ocean forces isotherms downwards, where they make contact with water masses with higher nutrient concentrations, thereby enhancing the nutrient flux to the deep source waters of the CCS. Increased winds and eddy activity further facilitate upward nutrient transport to the euphotic zone. However, the plankton community exhibits a complex and nonlinear response to increased nutrient input, as the food web dynamics tend to interact differently. This analysis highlights the difficulty in understanding how the marine ecosystem responds to a future warming climate, given to range of relevant processes operating at different scales.

## Introduction

Coastal upwelling systems, such as the California Current System (CCS), the Canary Current System, the Humboldt Current System, and the Benguela Current System, are among the most productive ecosystems in the global ocean^1,2^. In these systems, coastal upwelling is regulated by Ekman dynamics, where equatorward alongshore winds transport surface waters offshore, causing them to be replaced by cold and nutrient-rich waters from depth, thus increasing phytoplankton production. It has been suggested that eastern boundary current systems cover less than 2% of the global ocean surface, but contribute ~7% of the global primary production and more than 20% of the global fish catches^2^.

Evidence suggests that global phytoplankton biomass and productivity in the ocean have changed over time. At large scales, most observations and numerical models suggest that average phytoplankton biomass and primary productivity have declined over the past a few decades, and will to continue during the next century^3–6^. One explanation for this declining trend is that ocean surface warming increases upper-ocean stratification, which indirectly affects phytoplankton growth by limiting nutrient supply to the sunlit layer^3,7^. Another direct impact of increasing temperature on marine ecosystems is that the metabolic rates of both phytoplankton and zooplankton will increase, while heterotrophic processes are more sensitive to temperature than autotrophic processes^8^. This causes higher grazing rates by zooplankton and consequently a decrease in phytoplankton biomass under warming conditions^8,9^.

At regional scales, especially in coastal upwelling systems, the ecosystem response to surface warming becomes more complex. It is hypothesized that global warming will enhance land–sea temperature gradients that in turn will increase upwelling favorable winds (i.e., the Bakun hypothesis)^10^. Consistent with the Bakun hypothesis, future predictions indicate that such changes are also latitude-dependent and likely to change the spatial heterogeneity of coastal upwelling, with increased upwelling intensity and duration at higher latitudes^11^. Other studies also suggest alternative mechanisms, whereby poleward migration and intensification of major atmospheric high-pressure cells are found to drive intensified coastal upwelling^12,13^. Although the driving mechanism is still being actively debated, previous studies appear to consistently predict that the coastal upwelling in eastern boundary current systems has intensified and that the increasing trend will continue^14,15^. Change in upwelling is only one driving factor that regulates marine ecosystems. Changes in nutrient concentrations and nutrient ratios have been observed in the upwelling source waters of the CCS^16,17^, which are likely to affect both phytoplankton biomass and compositions. Moreover, mesoscale eddies in the CCS have been suggested to play an important role in transporting nutrients and phytoplankton^18^. Most of the model-based future predictions are too coarse to resolve these mesoscale features or detailed shelf dynamics. Considering that surface warming can otherwise increase upper-ocean stratification and decrease nutrient supply^19^, exploring the response of ecosystems to intensified upwelling and ocean surface warming would require a more detailed modeling framework in which physical processes, and nutrient and plankton dynamics can be evaluated.

In this study, a high-resolution (7 km in the horizontal) coupled physical–biological model was constructed and explored. The physical model was based on the Regional Ocean Modeling System (ROMS)^20^ and the biological model was based on the Carbon, Silicate, and Nitrogen Ecosystem model (CoSiNE-31)^21^ that includes 31 chemical and biological variables considering multiple plankton functional groups and multiple nutrient forms. The coupled model was dynamically downscaled from the climate simulated by an earth system model over the CCS region from 1970 to 2049 under the representative concentration pathway 8.5.

## Results

### Model evaluation

**Figure 1:**
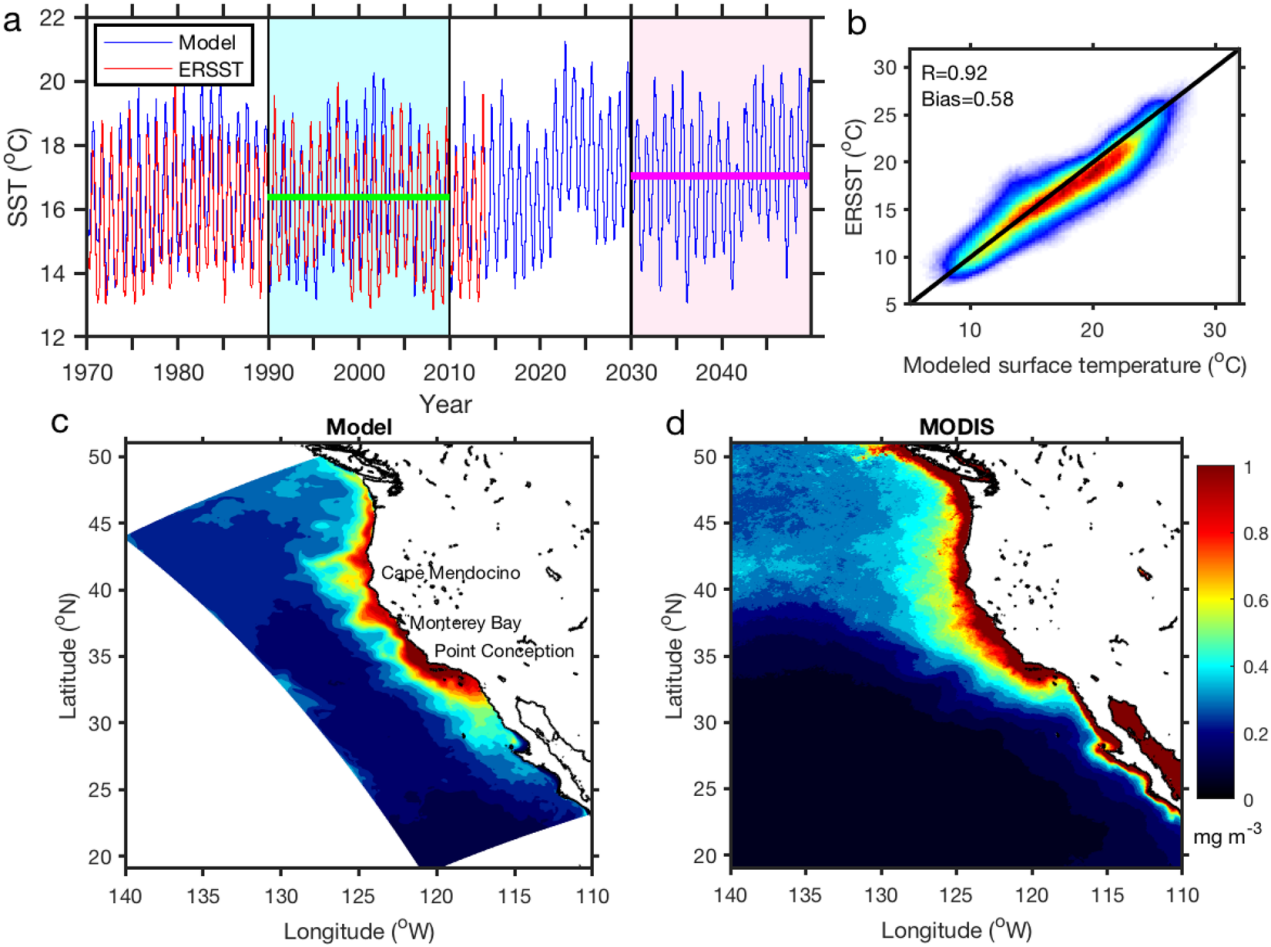
Model–data comparisons. (**a**) Modeled temperature (blue curve) in the surface layer at an offshore location (127°W, 35°N) compared with the ERSST dataset (red curve) from 1970 to 2013. Two 20-year periods are chosen for later comparison (period 1: 1990–2009; period 2: 2030–2049). The green and magenta lines denote the mean values of the two periods. (**b**) Scatter plot of the modeled surface temperature and the ERSST product (color showing data density) over the whole model domain between 1970 and 2013. (**c**) Modeled surface chlorophyll concentration (mg m^−3^) averaged for the upwelling season during 2002–2013. (**d**) MODIS observed surface chlorophyll concentration (mg m^−3^) averaged for the upwelling season during 2002–2013.

We first use two relatively independent variables to evaluate the performance of the coupled model with historical and present-day data. The modeled sea surface temperature (SST) at an offshore location (127°W, 35°N) is relatively consistent with the extended reconstructed sea surface temperature (ERSST) dataset from 1970 to 2013 (Fig. 1a), with a correlation coefficient of 0.87 (p < 0.01). The scatter plot for the whole model domain shows a robust correlation with a coefficient of 0.92 (p < 0.01). There is a mean bias of 0.58 °C between the modeled SST and the ERSST, which could also be attributable to the under-representation of key processes in the coarse ERSST dataset. To quantify future changes relative to the present day, two 20-year periods are compared, by which the systematic model bias can be removed^22^. The first period represents the present day (period 1: 1990–2009) and the second represents the future (period 2: 2030–2049). The averaged SST for the offshore location is 16.4 °C in period 1, increasing to 17.1 °C in period 2, with a warming rate of 0.018 °C yr^–1^.

The biological model reproduces a similar spatial pattern of surface chlorophyll concentration to that of remote sensing data from the Moderate Resolution Imaging Spectroradiometer (MODIS) (Fig. 1c and d). There is a strong cross-shelf gradient in chlorophyll, with more phytoplankton biomass onshore and associated with the coastal upwelling process. The model appears to underestimate surface chlorophyll in the very coastal region compared with the MODIS data. With the 7-km resolution, the model can resolve mesoscale features such as upwelling and eddies, but it fails to reasonably reproduce submesoscale and small-scale features such as coastal fronts. In the model, only major rivers are included. Line-source inputs of freshwater and nutrients via small streams at the continental margin are not represented, which might lead to the underestimation of phytoplankton chlorophyll. In addition, previous study has shown that the MODIS data are generally biased in regions with chlorophyll concentration higher than 1.0 mg m^−3^ due to the poor retrieval of remote sensing reflectance^23^.

### Future nutrient change and driving mechanisms

**Figure 2:**
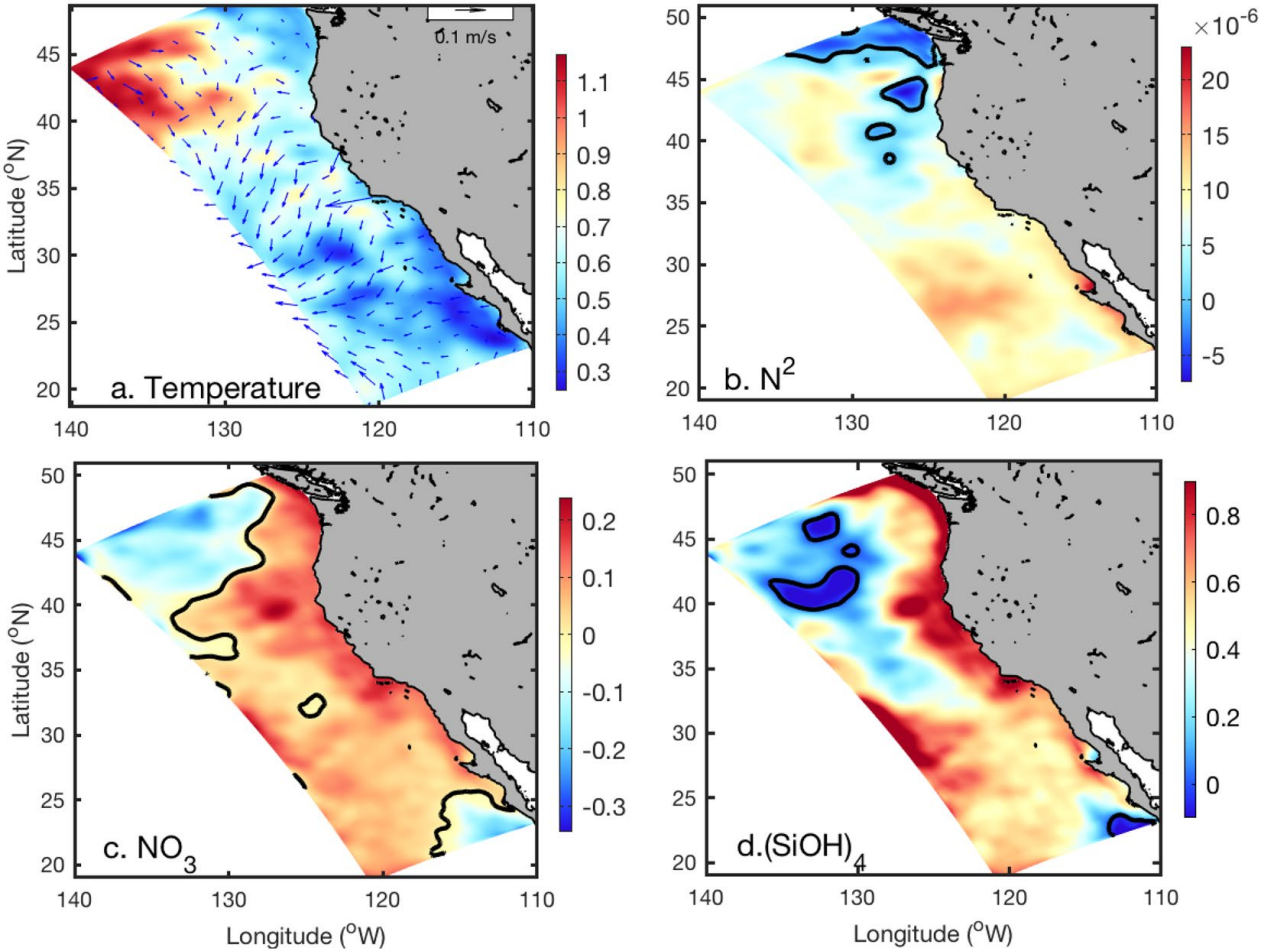
Spatial distributions in the difference in mean conditions between the two periods (period 2 − period 1) averaged over the upper 100 m. (**a**) Temperature (°C), with the mean surface current during period 1 superimposed. (**b**) Buoyancy frequency (s^−2^). (**c**) Nitrate (NO_3_, mmol-N m^−3^). (**d**) Silicate (Si(OH)_4_, mmol-Si m^−3^). Black contours in the figures denote the zero isoline.

To illustrate future changes, we compare the 20-year mean of physical and biological variables between the two periods (i.e., period 1 and period 2) (Fig. 2). The temperature increase in the upper 100 m occurs over the entire CCS, although there is strong spatial variability. For example, large increases are found in the northwestern corner of the domain, over 1000 km offshore from the Oregon coast, and relatively small increases are found close to the coast of Baja California (Fig. 2a). Buoyancy frequency, defined as ${N}^{2}=-(g/{\rho }_{0})\partial {\rm{\rho }}/\partial z$ (where *g* is acceleration due to gravity and ${\rho }_{0}$ is the reference density), is used to provide a quantitative measure of water column stratification. In response to surface temperature increases, most of the CCS region becomes more stratified, as illustrated by the increased buoyancy frequency over the upper 100 m. This increase in stratification could potentially decrease the upward transport of nutrients by vertical mixing (Fig. 2b). However, enhanced nitrate concentrations (NO_3_) are observed over the CCS, from Vancouver Island to Baja California, with significant increases in coastal regions (Fig. 2c). Coincident with the temperature increase in the northwestern corner of the domain, nitrate concentrations are predicted to decrease but with a relatively small magnitude. Other nutrients, such as silicate (Si(OH)_4_), show similar spatial patterns to the nitrate, but with larger amplitude (Fig. 2d).

**Figure 3:**
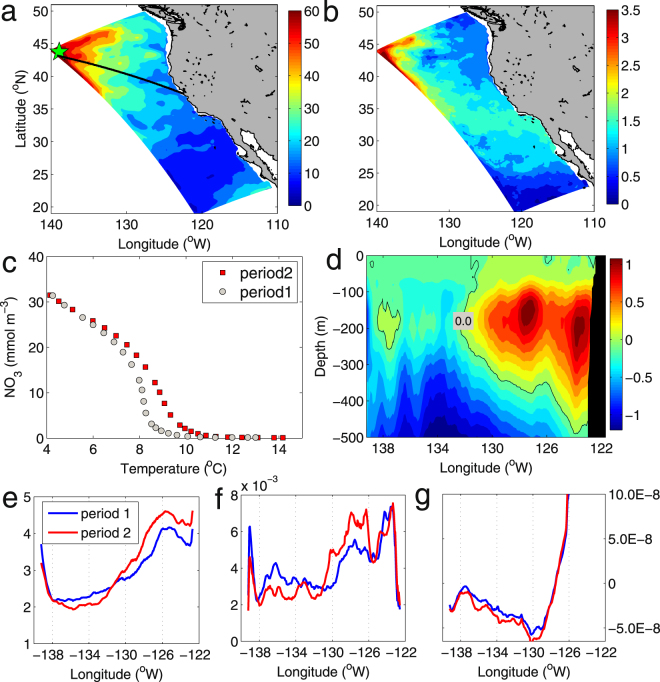
Distributions of deep nutrient changes and forcing terms along a cross-section. (**a**) Changes in isopycnal (26.4 kg m^−3^) depth (m) (period 2−period 1). (**b**) Changes in nitrate concentrations (mmol-N m^−3^) on the 26.4 isopycnal. (**c**) Temperature–NO_3_ relationship for the location denoted by the green asterisk in (**a**). (**d**) Vertical distribution of nitrate changes (mmol-N m^−3^) along a cross-section denoted by the black line in (**a**). (**e**) Nitrate concentration (mmol-N m^−3^) at 200 m depth along the cross-section for the two periods. (**f**) Eddy kinetic energy (EKE, m^2^ s^−2^) along the cross-section for the two periods. (**g**) Wind stress curl (N m^−3^) along the cross-section for the two periods.

The enhanced upper-ocean stratification induced by surface warming is not the dominant factor driving future changes in nutrients through vertical mixing processes. Although reduced nutrients are found in the northwestern corner of the domain, ~1500 km offshore from the Washington coast, associated with the largest temperature increase, the change in buoyancy frequency in that region is not significantly different from in other regions. To elucidate the mechanisms leading to changes in nutrient concentrations, we examined the spatial distribution of nutrients on different isopycnals below the mixed layer, as water mass transport at depth generally occurs along isopycnal surfaces. Surface warming results in an increase in upper water temperature that inevitably deepens isopycnals. As surface warming itself does not change the vertical distribution of nutrients, the deepening of isopycnals is thus associated with elevated nutrients for a particular isopycnal. Typically, the deeper the isopycnal becomes, the higher the nutrient concentration. This suggests that the deepening of the 26.4 isopycnal, generally located at a depth of 200 m, is unevenly distributed throughout the CCS (Fig. 3a). The northwestern corner of the domain, through which the North Pacific water enters the CCS (Fig. 2a), shows the strongest deepening of the 26.4 isopycnal: ~60 m compared with ~15 m in the offshore region of the central CCS. This spatial heterogeneity of isopycnal deepening creates a positive nutrient flux from the North Pacific into the CCS, which results in increased nutrients in the deep waters of the CCS, potentially available for the upwelled water along the coast (Fig. 3b). The nutrient flux to the CCS tends to be weaker for isopycnals shallower than 26.4 and becomes insignificant at depths immediately below the mixed layer. We propose that the isopycnal deepening is due to surface warming rather than large-scale upwelling and downwelling, as there is a good correlation with the spatial pattern of temperature increase. Moreover, as upwelling and downwelling move a water mass vertically without changing its properties, one would not expect significant changes in nutrients along un-outcropped isopycnals. This view is supported by the change in the nitrate–temperature relationship (Fig. 3c). For example, strong changes are found mostly in mid-depth waters (i.e., 100–400 m), consistent with previous studies^24^.

Given that nutrient levels are enhanced in the deep source waters of the CCS, we further examined changes in the mechanisms that transport subsurface nutrients to the sunlit layer. A cross-section oriented approximately parallel to water parcel trajectories into the CCS depicts strong increases in nitrate concentrations at ~100–400 m in the CCS, and decreases in the northwestern corner of the domain where the North Pacific water enters (Fig. 3d). At the large scale, nutrient concentrations in the subtropical North Pacific are predicted to decrease due to increased stratification, as suggested previously^16^. The present simulation suggests that the nutrient decrease in the northwest is more significant below 300 m (Fig. 3d), indicating the effect of local processes. The cross-section shows a correlation at depth between the changes in nitrate levels and mesoscale eddy activity, denoted by the eddy kinetic energy (EKE) (Fig. 3e and f). Oceanic eddies can perturb the ocean mean state and facilitate vertical exchange between surface and deep layers. To the west of roughly 131°W, the reduced nitrate concentration is associated with decreased EKE, as well as a suppressed negative wind stress curl, which result in enhanced downwelling in this region (Fig. 3g). To the east of 131°W, the increased nitrate concentration tends to follow increased EKE, and the wind stress curl shows no significant change.

**Figure 4:**
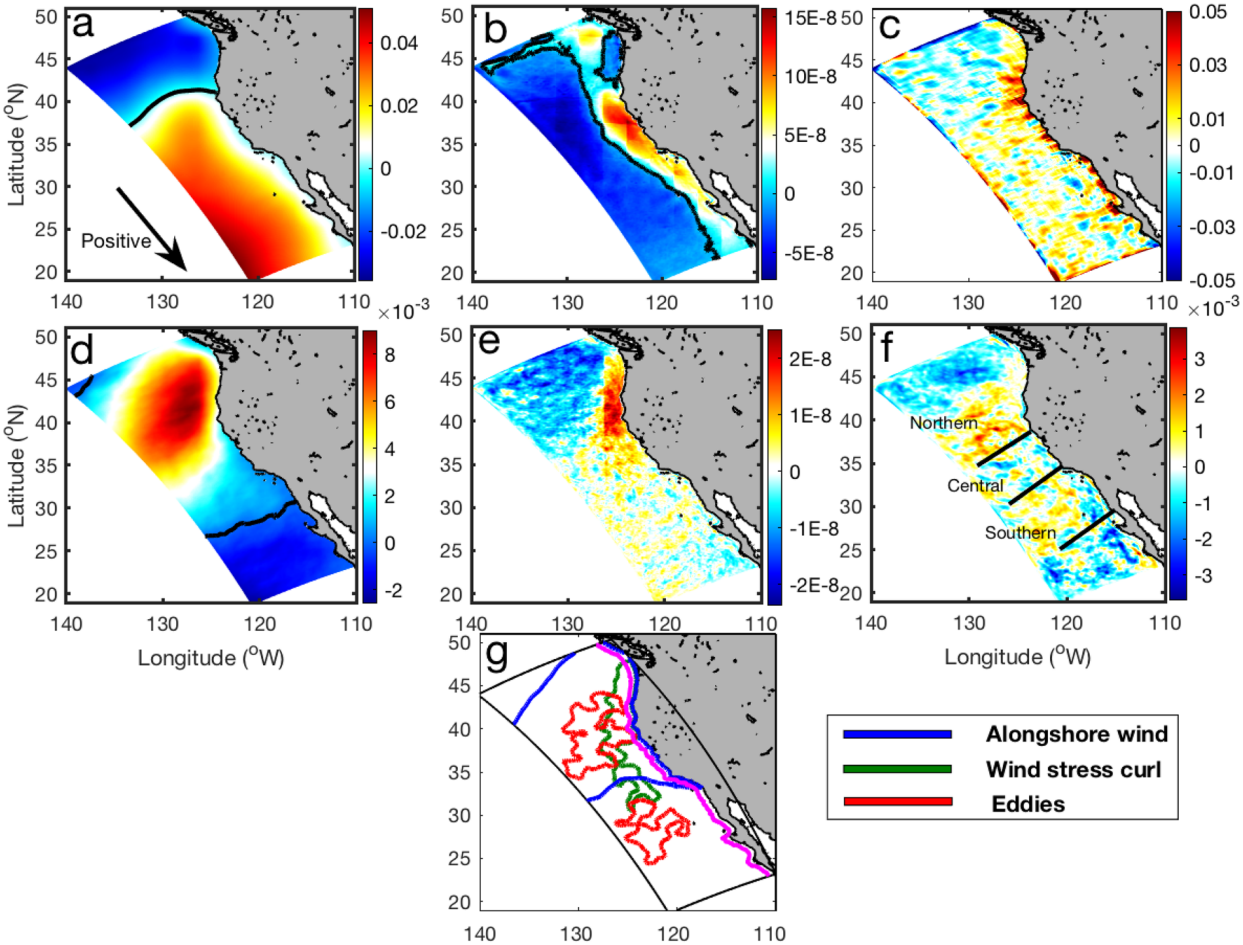
Spatial distributions of alongshore wind stress, wind stress curl, and EKE. (**a**) Alongshore wind stress (N m^−2^) averaged during period 1. (**b**) Wind stress curl (N m^−3^) averaged during period 1. (**c**) Vertical velocity (m d^−1^) changes (period 2−period 1) averaged over the upper 100 m. (**d**) Alongshore wind stress changes. (**e**) Wind stress curl changes. (**f**) EKE changes. (**g**) Regions with changes of >10% that promote upward water motion. Magenta line indicates a distance of 50 km from the shore.

Variable changes in the wind stress curl, alongshore wind, and EKE are evident for different regions within the CCS, indicating that a range of mechanisms are responsible for transporting deep nutrients to the sunlit layer (Fig. 4). The alongshore wind, which is particularly important in driving coastal upwelling via Ekman dynamics, shows a strong increase in regions off the coast of northern and central California, and a decrease in regions off Baja California (Fig. 4a and d). The increase in alongshore winds over the northern region reduces the coastal downwelling there, whereas the increase in alongshore winds over the central region enhances coastal upwelling, but both facilitate an upward nutrient flux. In contrast, the decrease in alongshore winds over the southern region tends to favor coastal downwelling. The wind stress curl, which induces local upwelling and downwelling with a smaller magnitude compared with alongshore winds, is generally positive along the coast and negative farther offshore (Fig. 4b). The model predicts a strong increase in wind stress curl over a region extending from the coast of northern California to the offshore region of central California, where the alongshore wind also increases (Fig. 4e). Consequently, the upward vertical velocity along the coast of central California increases (Fig. 4c). The changes in vertical velocity along the coast of central California are significantly correlated with the alongshore wind (correlation coefficient of 0.45), but show no relationship with the wind stress curl, especially for near-shore areas off central California.

In addition to wind changes, eddy activity increases under future conditions, particularly the offshore region (Fig. 4f). We identified regions where the changes are greater than 10% and promote upward water motion (Fig. 4g). The alongshore wind changes are most pronounced off central and northern California for both coastal and offshore regions, though the alongshore wind in the offshore region does not necessarily produce upwelling. The changes in wind stress curl are most pronounced in the coastal region of northern California and the offshore region of central California. Elevated eddy activity is observed in the offshore region of northern and southern California. Together, these three mechanisms drive increased vertical nutrient flux in both coastal and offshore regions. Interestingly, the ‘hot spot’ off Cape Mendocino, which shows the strongest nutrient increase, is located close to where the effects of the three mechanisms are combined (Fig. 2c).

### Future biological change

**Figure 5:**
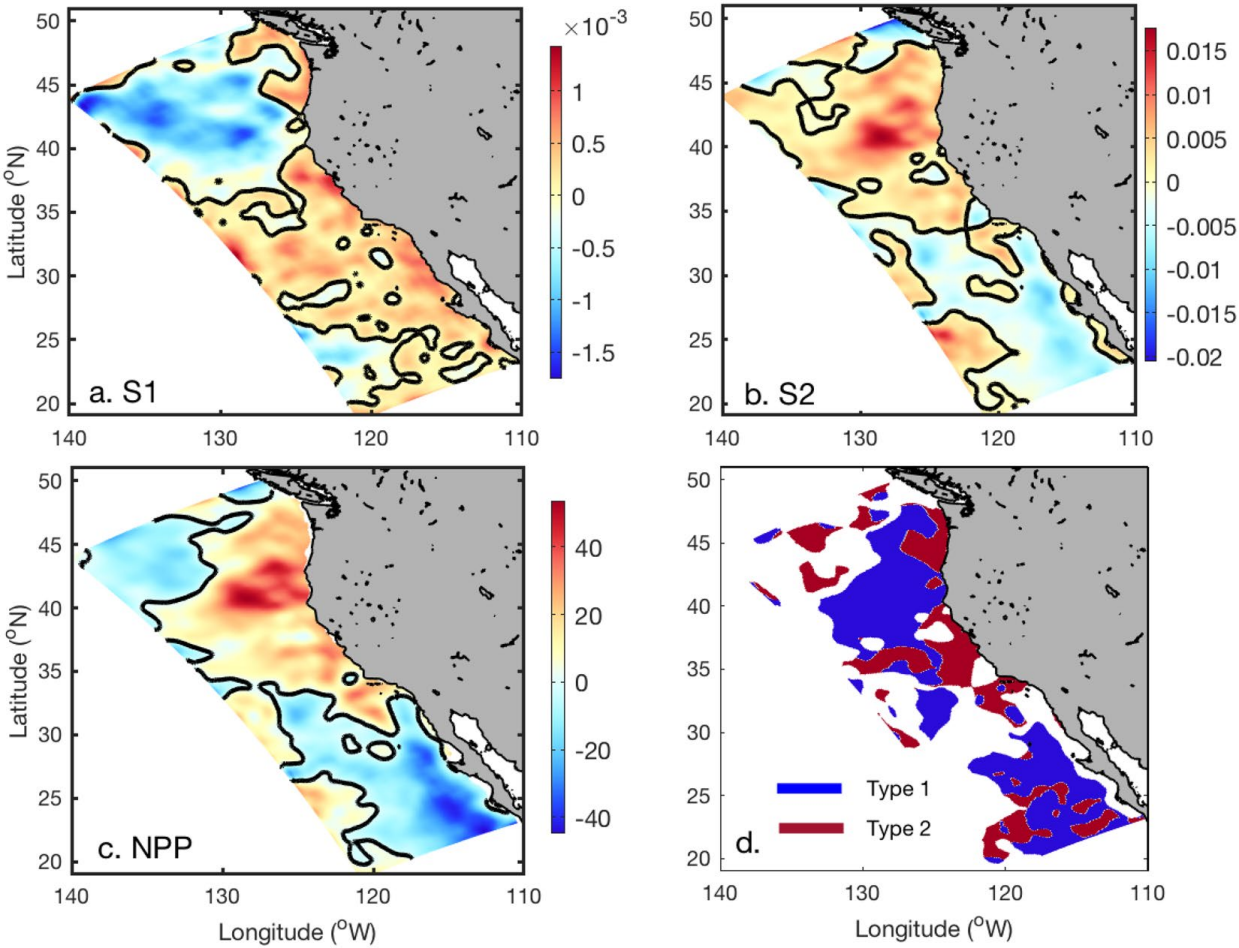
Spatial distributions of the difference in mean conditions between the two periods (period 2−period 1) averaged over the upper 100 m. (**a**) Small phytoplankton (S1, mmol-N m^−3^). (**b**) Diatoms (S2, mmol-N m^−3^). (**c**) Depth-integrated (0–100 m) net primary production (NPP, mg C m^−2^ d^−1^). (**d**) Spatial distributions of the two types of ecosystem change. In type 1, changes in S1 are in the opposite direction to changes in S2, ZZ1, and ZZ2. In type 2, changes in S1, S2, ZZ1, and ZZ2 are all in the same direction. Black contours in the figures denote the zero isoline.

The consequences of nutrient change vary with each phytoplankton functional group, and we generally find opposite trends for changes in small phytoplankton (S1) and diatoms (S2) in the northern and southern CCS (Fig. 5). Small phytoplankton show consistent decreasing trends in biomass in offshore regions of the northern CCS, and increasing trends in most of the regions of the central and southern CCS. The spatial pattern of change in the biomass of diatoms is generally opposite to that of small phytoplankton in the northern and southern CCS. In the central CCS, however, both small phytoplankton and diatoms are predicted to increase. Although the spatial patterns are clear, the magnitude of the changes in small phytoplankton are relatively small, accounting for only about 5% of present-day values. For diatoms, however, relative changes are greater than 10% over most of the domain, especially in offshore regions where the biomass of diatoms is generally low under present-day conditions. This results in a similar increasing spatial pattern of total net primary production (NPP) to diatoms, where large increases occur in northern and central regions of the CCS, and large decreases occur offshore of the CCS in the south (Fig. 5c).

The contrasting patterns of change in phytoplankton biomass and nutrients in the CCS reflect modulations associated with zooplankton grazing dynamics. The spatial patterns of changes in S1, S2, microzooplankton (ZZ1), and mesozooplankton (ZZ2) generally reveal two types of ecosystem interaction mechanisms (Fig. 5d). Type 1, which is mostly observed in offshore regions, is associated with food web dynamics in which changes in S1 are opposite to changes in S2, ZZ1, and ZZ2. Specifically, increases in S2 can increase the biomass of ZZ2 due to enhanced grazing, which in turn can shift and reduce the grazing pressure on ZZ1 from ZZ2, thereby leading to an increase in ZZ1 biomass. The increases in ZZ1 then exert increased grazing pressure on S1 and reduce S1 biomass^25^. This energy flow also works in the opposite direction. Type 2, which is generally observed in the coastal regions off northern and central California and some deep-water regions, is associated with bottom-up ecosystem dynamics in which changes in S1, S2, ZZ1, and ZZ2 are all in the same direction (i.e., all increasing or all decreasing). For this type of response, changes in phytoplankton and zooplankton are tightly linked with nutrient changes, whereas both nutrients and grazing are important factors controlling phytoplankton distributions for type 1. Although changes in surface light that might influence phytoplankton photosynthesis are also observed, sensitivity studies suggest that this is a minor factor compared with changes in nutrients and grazing in terms of regulating biological structures.

## Discussion and Conclusion

The above analysis indicates that both deep nutrients and upwelling are key factors in driving an upward nutrient flux to the euphotic zone in the CCS. Furthermore, water mass transport and nutrient flux budgets in a region off the coast of central California, extending from the coast to 800 km offshore, show that the upwelling intensity at 200 m depth is predicted to increase by 28.2% in period 2 and the nitrate concentration by 5.8%. This highlights the relatively important role of changes in local processes. The increasing rate of nutrients is in agreement with recent findings^17^. When the budget calculation is limited to an offshore region located 150–800 km from the coast, the change in upward velocity change is 15.7% and the change in nitrate concentration is 5.6%. This implies that coastal upwelling is important in regulating the nutrient flux in the CCS. A previous modeling study predicted less change in vertical velocity compared to that found here^16^, probably due to the present model’s ability to fully resolve coastal dynamics, as the previous study used a rather coarse earth system model.

There are three major drivers leading to changes in vertical velocity, and their relative contributions vary in different regions (Fig. 4g). In the coastal region (<150 km) of the central CCS, the vertical velocity at 100 m depth will increase about 20%, which is largely caused by the increase of the alongshore wind (~28%). In the offshore region of the central CCS (150–800 km), the change of vertical velocity at 100 m is predominantly contributed by the change of wind-curl-induced Ekman pumping (~92%). In the offshore region of the southern CCS, this contribution drops to about 16%. Mesoscale eddies and other processes dominate the change of vertical velocity in this region. A previous study has shown that mesoscale eddies tend to decrease nutrient concentrations in the upwelling region through offshore transport^18^. Our findings are consistent with this process, as the offshore region was the site of greatest change in mesoscale eddy activity.

The nutrient increase at depth is also consistent with observed decreasing oxygen in other regions, which is largely attributed to increased stratification, reduced ventilation, and increased coastal production^26–28^. In the CCS, the advection of low-oxygen waters into the region has been suggested to contribute to the observed oxygen decline^27^. The water mass with increased nutrient amounts transported to the upwelling region due to warming is generally accompanied with low oxygen levels. With increased nutrients transported to the CCS and enhanced coastal upwelling, the oxygen level is likely to show a significant decline, leading to an increase in hypoxic events in the future.

**Figure 6:**
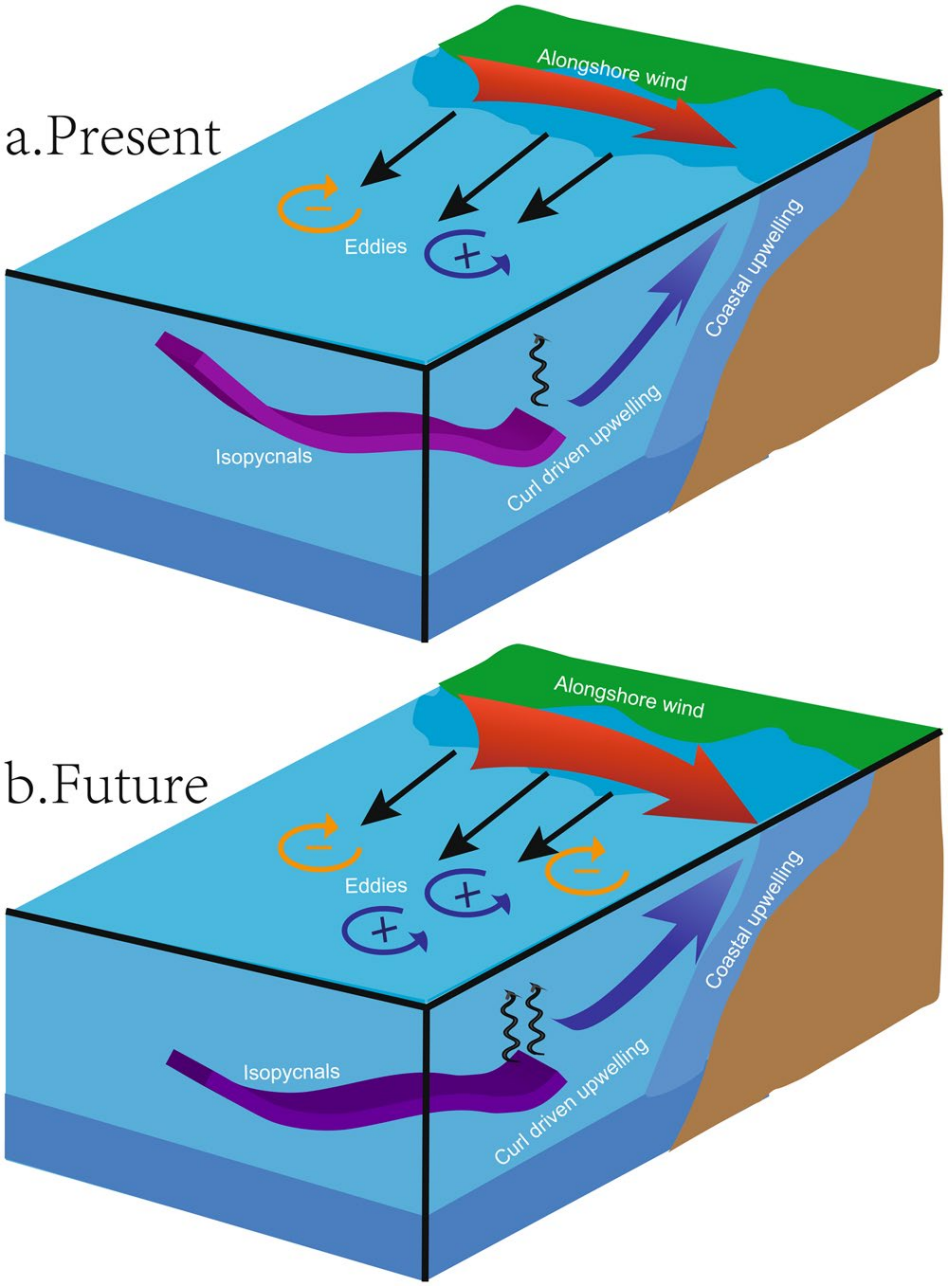
Conceptual diagram of future changes in deep nutrients, alongshore winds, wind stress curl, coastal upwelling, and eddy activity. In the future, differences in isopycnal deepening between the gyre and coastal regions mean that more nutrients are transported to the CCS. Enhanced local alongshore winds promote coastal upwelling, and changes in mesoscale eddies and wind stress curl promote upward nutrient flux in the offshore region.

Overall, the model prediction depicts a picture of how ecosystem processes in the CCS will respond under future climate conditions, including changes in the nutrient concentrations of deep source waters and in the physical mechanisms responsible for the upward transport of nutrient-rich waters to the sunlit layer (Fig. 6). Moreover, the dynamical downscaling of both the physics and biogeochemistry over the CCS sheds light on the fact that increased nutrient concentrations do not linearly correspond to increases in plankton biomass that are further controlled by ecosystem food web dynamics (e.g., phytoplankton and zooplankton interactions). Our results highlight the importance of the difference in warming between gyre and coastal regions in providing a mechanism driving a positive nutrient flux to the coastal region. Local dynamics from upwelling favorable winds and mesoscale eddies, which are generally not well represented in earth system models, have been identified as key factors affecting future ecosystem change. Although here we only focus on future responses of the CCS, our study provides a dynamical framework that may help to better understand how other coastal upwelling ecosystems are likely to respond to future climate change.

## Methods

We focus on coastal upwelling in the CCS region using a high-resolution coupled physical–biological model that was specifically developed to assess the potential changes associated with anthropogenic climate change and their impacts on the ecosystem. The physical model is based on the Regional Ocean Modeling System (ROMS) and the model domain covers the region 18.5–50.5°N and 110–140°W. The horizontal resolution is ~7 km and the model uses 50 vertical levels in terrain-following sigma-coordinates, weighted towards the surface to better resolve the mixed layer. The biological model is based on the Carbon, Silicate, and Nitrogen Ecosystem model (CoSiNE-31) that includes 31 chemical and biological variables considering multiple plankton functional groups and multiple nutrient forms. In the CoSiNE-31, small phytoplankton (S1) are typically defined with a size of <5 $\mu {m}$ in diameter and are easily grazed by microzooplankton (ZZ1), with daily net productivity being largely remineralized^29^. Diatoms (S2) grazed by mesozooplankton (ZZ2) are relatively large phytoplankton (>5 $\mu m$ in diameter) that have the potential to grow rapidly under optimal nutrient conditions^30,31^.

The coupled model was forced at the surface and open boundaries with the Geophysical Fluid Dynamics Laboratory Earth System Model (ESM2M)^32–34^. Following the Coupled Model Intercomparison Project Phase 5 (CMIP5) design protocol, historical forcing is applied prior to 2005 and the representative concentration pathway 8.5 (RCP8.5)^35^ forcing is used after 2006. The high-resolution ROMS–CoSiNE coupled model was used to dynamically downscale the climate simulated by the earth system model over the CCS region from 1970 to 2049.

The extended reconstructed sea surface temperature (ERSST v3b) derived from the international comprehensive ocean–atmosphere dataset on a 2° × 2° grid was used to compare with modeled sea surface temperature (SST)^36^. Monthly averaged chlorophyll concentrations (horizontal resolution of 4 km) were obtained from the Moderate Resolution Imaging Spectroradiometer (MODIS) (http://oceancolor.gsfc.nasa.gov/)^37^ and used as a metric to assess modeled phytoplankton biomass near the surface. Figures 1–4 in this paper were produced using MATLAB R2017a (http://www.mathworks.com).
